# The Latest Crusade against Homœopathy

**Published:** 1893-06

**Authors:** 


### HOMŒOPATHIC PHYSICIAN,

A MONTHLY JOURNAL OF

HOMŒOPATHIC MATERIA MEDICA AND CLINICAL MEDICINE.

"If our school ever gives up the strict inductive method of Hahnemann, we are lost, and deserve only to be mentioned as a caricature in the history of medicine."—CONSTANTINE HERING.

Vol. XIII. JUNE, 1893. No. 6.

### EDITORIAL.

THE LATEST CRUSADE AGAINST HOMŒOPATHY.—Some surprise has been manifested by certain subscribers to this journal that no notice has been taken by its editor of the $100 prize offered for the best essay against the "fallacies" (?) of Homœopathy.

All the other journals in the homœopathic ranks have from time to time made comments or replies more or less severe to the attacks of the originator of the prize, this rather young "knight of the lancet" who seeks to impale Homœopathy upon the point of his pen.

THE HOMŒOPATHIC PHYSICIAN alone has remained conspicuously silent.

The truth is, the treatment of the case of the aforesaid knight at the hands of the other editors has been so clever and so thorough that there is apparently nothing more to be said.

Still, as expression of opinion is desired by a few of our readers, it may be well to say that an attack which is so evidently conceived in malice, that shows so plainly its author's determination not to learn any of the facts of Homœopathy, that seems to have been used as a personal advertisement of its author, hardly deserves notice. It is not worth answering.

For the comfort of those who may have been rendered uneasy by these attacks, we may remind them of the oft-quoted story of Gallileo, who invited the persecuting monks to look through the telescope and behold with their own eyes, and yet they refused so plain an opportunity to learn the truth.

The very first proposition of Hahnemann, that nothing can be known of the nature of drugs in relation to the human system except by trying them upon the healthy, seems so obviously true and reasonable that it is a matter of surprise any one could be found to dispute it. Yet it has been so disputed, and vehemently, by physicians of the old school of medicine. Nevertheless, in late years they have been imitating the homœopathists in that particular, though in a very crude and ineffectual way.

Nothing could be more in harmony with or more nearly parallel to the lines along which physics and chemistry, as well as the other natural sciences, have made their remarkable progress than this same proposition that drugs must first be proved upon the healthy in order that we may get their pure effects, free from the obscuring phenomena of disease. Rigorously following out such a plan would at once reduce to the lowest point the sources of doubt and error in judging of the phenomena observed when drugs are administered to the sick. It is the plan of the physicist, the chemist, the naturalist, in the laboratory. When the scientific investigator would explain in a rational way any natural phenomenon, he considers all the factors likely to enter into the production of that phenomenon. He seeks to exclude as many of these influences as possible, so that he may have, if possible, only one of them to observe at a time. He is then able to draw a reliable conclusion as to the nature of the phenomenon and its cause.

Thus apparatus must be invented to perform the experiments and according to the difficulty found in excluding disturbing influences becomes the necessity of devising variations in the structure of the apparatus, and thus it becomes complicated. Here then is the explanation of the elaborate and varied character of instruments used in experimental research—that in the investigation of a phenomenon, the problem is simplified by the exclusion of as many confusing factors as possible in order that the attention may be fixed upon the actions of one and its agency accurately determined. This is what the use of apparatus means. This, too, is what Hahnemann meant when he proposed to try the effects of drugs upon people who were as nearly healthy as possible.

But the instruments and apparatus of the laboratory catch the eye and compel admiration and wonder by their beauty and complexity, and so their manipulator escapes persecution, and gains instead admiration. Hahnemann's method, on the other hand, does not deal with these instruments, it does not appeal to the senses, its detail is inconvenient of application, the results difficult to determine, and so it attracts none, and brings only censure to its author.

Now it would seem that any mind that is so superficial that it cannot or does not perceive the perfect parallelism between the method of Hahnemann and that of the most distinguished investigators and discoverers, and is unwilling to admit the scientific exactitude in the one case as well as in the other, is unworthy of a reply or to our engaging in the controversy he seeks to provoke.

We may stop here. This first proposition of Hahnemann, obviously scientific as it is, having been persistently repudiated by our enemies, it will not be necessary to consider any of the other propositions in Hahnemann's grand discovery, especially as they are so well known to the earnest readers of these pages who have inspired the foregoing remarks.

